# Structural and functional aspects of the interaction partners of the small heat-shock protein in *Synechocystis*

**DOI:** 10.1007/s12192-018-0884-3

**Published:** 2018-02-23

**Authors:** Erik G. Marklund, Yichen Zhang, Eman Basha, Justin L. P. Benesch, Elizabeth Vierling

**Affiliations:** 1 Department of Chemistry, Physical & Theoretical Chemistry Laboratory, University of Oxford, Oxford, OX1 3QZ UK; 2 Department of Chemistry – BMC, Uppsala University, Box 576, Uppsala, 75123 Sweden; 3 Department of Biochemistry & Molecular Biology, University of Massachusetts, Amherst, MA 01003 USA; 4 Present Address: Alorica, Inc., Irvine, CA USA; 5 Present Address: Department of Molecular and Cellular Biology, University of Arizona, Tucson, AZ 85721 USA

**Keywords:** Small heat-shock protein, α-Crystallins, Molecular chaperone, Cyanobacteria, Bioinformatics

## Abstract

The canonical function of small heat-shock proteins (sHSPs) is to interact with proteins destabilized under conditions of cellular stress. While the breadth of interactions made by many sHSPs is well-known, there is currently little knowledge about what structural features of the interactors form the basis for their recognition. Here, we have identified 83 in vivo interactors of the sole sHSP in the cyanobacterium *Synechocystis* sp. PCC 6803, HSP16.6, reflective of stable associations with soluble proteins made under heat-shock conditions. By performing bioinformatic analyses on these interactors, we identify primary and secondary structural elements that are enriched relative to expectations from the cyanobacterial genome. In addition, by examining the *Synechocystis* interactors and comparing them with those identified to bind sHSPs in other prokaryotes, we show that sHSPs associate with specific proteins and biological processes. Our data are therefore consistent with a picture of sHSPs being broadly specific molecular chaperones that act to protect multiple cellular pathways.

**Electronic supplementary material:**

The online version of this article (10.1007/s12192-018-0884-3) contains supplementary material, which is available to authorized users.

## Introduction

Small heat-shock proteins (sHSPs) are a diverse family of proteins that share a conserved ≈ 90-residue α-crystallin domain (ACD) that is flanked by variable N- and C-terminal regions (Basha et al. 2012; Hilton et al. 2013; McHaourab et al. 2009). Although sHSPs are relatively small as monomers (12 to 42 kDa), the majority assemble into large oligomers. These range in size from 12 to > 40 subunits, with some family members being monodisperse and others forming polydisperse ensembles (Basha et al. 2012; Hilton et al. 2013; McHaourab et al. 2009). Found in all kingdoms of life, many sHSPs have been demonstrated in vitro to act as ATP-independent molecular chaperones with the ability to capture denaturing proteins in a partially unfolded form such that they can be reactivated by the cell’s ATP-dependent chaperones. Recent reviews have described models for this canonical mechanism of sHSP chaperone action; however, details are derived primarily from in vitro studies with recombinant proteins and model interactors from non-homologous organisms (Haslbeck and Vierling 2015; Treweek et al. 2015). Thus, a major gap in our understanding of sHSP mechanism is the considerable lack of information about which substrates they protect in the cell.

In order to investigate the properties of proteins that are sHSP interactors, we identified HSP16.6 from the single-celled cyanobacterium *Synechocystis* sp. PCC 6803 (hereafter *Synechocystis*) as an ideal system to interrogate. HSP16.6 is the only sHSP in *Synechocystis* (Giese and Vierling 2002; Lee et al. 2000). It is strongly induced at high temperature, and cells deleted for HSP16.6 (Δ16.6) grow normally at optimal growth temperature but are sensitive to heat stress (Giese and Vierling 2002, 2004). The temperature-sensitivity phenotype of Δ16.6 cells has enabled studies of sHSP properties required for activity in vivo in a homologous system. Crucially, point mutations in the N-terminal domain were found to decrease heat tolerance in vivo, but to have no effect on the efficiency of chaperone function in assays with model substrates in vitro (Giese et al. 2005). This observation emphasizes the need to identify native interactors of sHSPs and renders *Synechocystis* an excellent system with which to do so.

We previously used immunoprecipitation and mass spectrometry (MS)-based proteomics to identify 13 proteins associated in vivo with HSP16.6 from *Synechocystis* cells that had been heat-stressed prior to cell lysis (Basha et al. 2004). Notably, these 13 proteins were not detected in equivalent pull-downs from cells that had not been heat-stressed, or when recombinant HSP16.6 was added to heat-stressed Δ16.6 cells before lysis (to control for sHSP-protein interactions that might occur in the lysate, as opposed to during heat stress in vivo). Although these proteins were associated with the sHSP in the soluble cell fraction, they were also found in the insoluble cell fraction after heat stress (Basha et al. 2004). All of these proteins, whose functions span a variety of cellular processes, including translation, transcription, secondary metabolism, and cell signaling, could be released from the immunoprecipitate by addition of DnaK, co-chaperones, and ATP (Basha et al. 2004). In addition, one of these interactors, a serine esterase, when purified, was shown to be heat sensitive and to associate with HSP16.6 and thereby be protected from insolubilization (Basha et al. 2004). While these data identified 13 proteins as potential interactors for canonical sHSP chaperone function, their relatively small number meant it was not possible to derive any common protein features that might dictate interaction with the sHSP.

Here, we have extended the identification of HSP16.6-interactors to a total of 83 proteins by performing an affinity pull-down from heat-stressed *Synechocystis*. By performing rigorous bioinformatic analyses, we provide new insights into the primary and secondary structural properties of proteins that interact with sHSPs in the soluble cell fraction during stress. We also catalogue the functions of the interactors and compare these to sHSP interactors previously identified in two other prokaryotes, *Escherichia coli* and *Deinococcus radiodurans* (Bepperling et al. 2012; Fu et al. 2013). Our combined results indicate that sHSPs protect a specific yet diverse set of proteins from aggregation in the cell.

## Methods

### Affinity isolation of HSP16.6-interacting proteins

Isogenic *Synechocystis* strains were used in which the wild-type HSP16.6 gene had been replaced with a spectinomycin resistance gene (*aadA* gene) (ΔHSP16.6 strain) or with the spectinomycin gene and HSP16.6 carrying a Strep-tag II affinity tag (WSHPQFEK) on the C-terminus (HSP16.6-Strep strain) (Basha et al. 2004). This HSP16.6-Strep strain had been shown previously to behave like wild type in assays of heat tolerance (Basha et al. 2004), and recombinant HSP16.6-strep protein was equivalent to untagged protein in assays of chaperone activity in vitro (Friedrich et al. 2004).

Cells were grown in 50-mL cultures at 30 °C as described previously to *A*_730_ ≈ 0.2 (Basha et al. 2004) and then subjected to treatment at 42 °C for 2 h followed by 1 h recovery at 30 °C, to allow accumulation of HSP16.6-Strep protein. Control samples were prepared directly after this treatment, while heat-stressed samples were treated for an additional 30 min at 46 °C. To control for interaction of HSP16.6-Strep protein during sample processing, recombinant HSP16.6-Strep protein was added to heat-stressed samples of the ΔHSP16.6 strain directly after heat treatment at a concentration matching that in heat-stressed cells. Cells were harvested, suspended in 1.5 mL lysis buffer (25 mM HEPES-KOH, 0.2 M NaCl, 0.5% Triton X-100, 5 mM ϵ-aminocaproic acid, 1 mM benzamidine, 1 μg mL^−1^ leupeptin, and 1 mM EDTA, pH 7.5), and opened as described previously (Basha et al. 2004). The soluble fraction was mixed with 30 μL of Strep-Tactin resin (Sigma) at 4 °C for 2 h. Resin was washed six times in lysis buffer, and bound proteins were eluted using either sample buffer (for SDS-PAGE) or isoelectric focusing (IEF) rehydration buffer (for 2D gels) (7.0 M urea, 2.5 M thiourea, 2% CHAPS, 2% IPG buffer pH 3–10 NL (Amersham Biotech), and 3 mg mL^−1^ dithiothreitol).

For 2D gel analysis, pH 3–10 NL first dimension strips (18 cm; Amersham Biotech) were rehydrated overnight at room temperature using 600 μL of sample in IEF rehydration buffer. IEF was carried out for 2 h at 150 V, 2 h at 300 V, 5 h at 500 V, and 7 h at 3500 V. The second dimension was separated by 11–17% SDS-PAGE for 30 min at 15 mA and then for 7 h at 25 mA. Samples were also separated by SDS-PAGE according to standard protocols, using 8% acrylamide gels in order to afford good separation of proteins above 100 kDa, which are typically not well resolved on the 2D system. Gels were silver stained according to a previous protocol (Rabilloud 2012)*.*

### Protein identification by means of mass spectrometry

Proteins unique to the heat-stressed HSP16.6-Strep sample were excised from 1D or 2D gels and digested with trypsin, and peptides were prepared for MS as described previously (Basha et al. 2004). Peptide extracts were introduced onto a 100-μm I.D. × 5-cm C18 column using an autosampler and separated with a 25-min gradient of 2–100% acetonitrile in 0.5% formic acid. The column eluate was directed into a Thermo Finnigan LCQ Deca ion trap mass spectrometer. The mass range scanned was 400 to 1500 *m*/*z*, and data-dependent scanning was used to select the three most abundant ions in each parent scan for tandem MS. Peptides were searched using SEQUEST and allowed for static modification of Cys (57 Da; iodoacetamidation), and differential modification of Met (16 Da; oxidation) was considered. X correlation cutoffs of 2.0 for 2+ ions, 3.0 for 3+ ions, and delta Xcorr > 0.05 were applied, and data were sorted using DTASelect (Tabb et al. 2002). The complete list of 83 proteins identified as HSP16.6 interaction partners from these and our previous experiments (Basha et al. 2004) is given in Supplemental Table 1. For the purpose of comparisons and calculations, this set is considered to represent sHSP interactors and denoted *I*, where |*I*| = 83. Known protein-protein interactions (PPIs) from yeast-2-hybrid experiments are available for *Synechocystis* (Sato et al. 2007). We identified all PPIs made by members of *I* (Supplemental Table 1), excluding PPIs that were not identified with multiple positive prey clones, in order to avoid false positives.

### Bioinformatic analyses

The *Synechocystis* sp. PCC 6803 genome (Kaneko et al. 1995; Kotani et al. 1995) was obtained from CyanoBase, http://genome.microbedb.jp/cyanobase/ (Nakamura et al. 1998). A set *G* representing the genome, containing all proteins such that *I* ⊆ *G*, was created from the protein-coding sequences in the genome. Only proteins with estimated isoelectric point (pI) within the range 4–9.5 and mass *m* between 10 and 200 kDa, corresponding to the range of proteins that could be identified in either the 1D or 2D gels, were included (see Supporting Information). This filtering resulted in *G* comprising 3021 proteins (i.e., |*G*| = 3021), which amounts to > 80% of the proteins encoded in the genome.

The mass, sequence length *n*_aa_, and abundance (absolute numbers *n*_*F*_ and frequencies *f*_*F*_ = *n*_*F*_/*n*_aa_) of various sequence features *F* were determined for every protein. These were DnaK-binding motifs; VQL, IXI, and [I/L/V]X[I/L/V] motifs (where X refers to any amino acid); charged (D,E,H,K,R), positive (H,K,R), negative (D,E), and hydrophobic (C,F,I,L,M,V,W) residues. DnaK-binding motifs were identified using a previously described algorithm (Van Durme et al. 2009), and the other motifs were found through *regexp* pattern-matching using the Python Standard Library. Long-range disorder was predicted with IUPred (Dosztanyi et al. 2005a, b) using default parameters, and residues with a score > 0.5 were considered unstructured. For the remainder, secondary structure was predicted from the sequences using the EMBOSS (Rice et al. 2000) implementation of the GOR method. β-strands and β-turns were pooled together into “β-structures.” Average abundances were calculated separately for *I* and *G*.

### Statistical significance testing and representation

A bootstrapping approach was employed to assess the statistical significance of any differences between *I* and *G*. First, a random subset, *R*, was taken from *G* by arbitrarily picking, with replacement, of 83 proteins (i.e., *R* ⊆ *G* and |*R*| = |*I*|). The mean, ${\overline{Q}}_R$, was then calculated for the given quantity of interest *Q*, to allow comparison with ${\overline{Q}}_I$, the mean calculated from *I* for the same quantity. This was repeated *N* times, after which the *p* value was calculated as the frequency by which ${\overline{Q}}_R\ge {\overline{Q}}_I$ or ${\overline{Q}}_R\le {\overline{Q}}_I$, in the respective cases of ${\overline{Q}}_I>{\overline{Q}}_G$ and ${\overline{Q}}_I<{\overline{Q}}_G$. For each quantity, a total of *N* = 100,000 iterations was run, and the statistical significance was tested at the 0.01 level.

Kernel density estimates were plotted for all quantities where a statistically significant difference was found. A Gaussian kernel with a bandwidth equal to 2% of the visible range was used in all cases and the amplitude was set such that the integrated density was equal to the number of proteins in each set. As such, the amplitudes are inversely proportional to the ranges along the *x*-axis, and their heights can thus differ substantially between distributions. Moreover, the *y*-axes’ ranges were chosen to make the *I* and *G* distributions occupy the same visible area in the resulting plot.

### Biological function analysis

A PANTHER Overrepresentation Test (release 20170413) against the GO Ontology database (release 20170926) was made for all proteins in *I*, using the *Synechocystis* reference list and the “GO biological process complete” annotation data set. Bonferroni correction was applied for multiple testing, and a *p* value cut-off of 0.05 was used to filter the results. Proteins that were not mapped to any entry in the reference list were added to the set of “unclassified” proteins. Enrichment was defined as *n*_*p*_/E(*n*_*p*_), where *n*_*p*_ is the number of proteins in *I* being ascribed to biological process *p*, and E(*n*_*p*_) is the expected number of such proteins based on their frequency in *G* and the size of *I*. Proteins that were assigned to the GO-class “biological process” but not to any of its subclasses were given the collective label “other biological process.” Since a single protein can have multiple classifications, the sum of proteins in the different classes exceeds |*I*|.

Protein BLAST (Altschul et al. 1990) was used to find orthologs among the interactors identified for HSP16.6 in *Synechocystis*, IbpB in *E. coli* (Fu et al. 2013), and HSP20.2 in *D. radiodurans* (Bepperling et al. 2012). Three pairwise comparisons were made to define the overlap between the sets of interactors, where the list of interactors from one organism was used as the “database” and the list of interactors from the other as the “query.” Using *E. coli* as the database yielded poorly annotated hits; hence, the primary database was set to be *Synechocystis* and the secondary database to be *D. radiodurans*. An *E* value cut-off of 10^−10^ was used for all BLAST searches, and whenever a protein in the query yielded several matches the one with the lowest *E* value was chosen. Lastly, the overlap between *E. coli* and *D. radiodurans* was used as a query against *Synechocystis* in order to find the overlap between the interactors in all three organisms. The triply overlapping set of proteins were also analyzed for an overrepresentation test in *Synechocystis* as described above, but without imposing a *p* value because of the small number of proteins in the query.

## Results

### Identification of proteins associated with HSP16.6 during heat stress in vivo

To identify a larger number of HSP16.6-associated proteins than we did previously (Basha et al. 2004), we developed a *Synechocystis* strain in which the wild-type *HSP16.6* gene was replaced with an *HSP16.6* gene modified to encode a Strep-tag II at the C-terminus. HSP16.6-Strep was shown to complement HSP16.6 in vivo in thermotolerance assays (Basha et al. 2004), as well as functioning in vitro to protect model interactors from irreversible heat-denaturation (Friedrich et al. 2004).

**Fig. 1:**
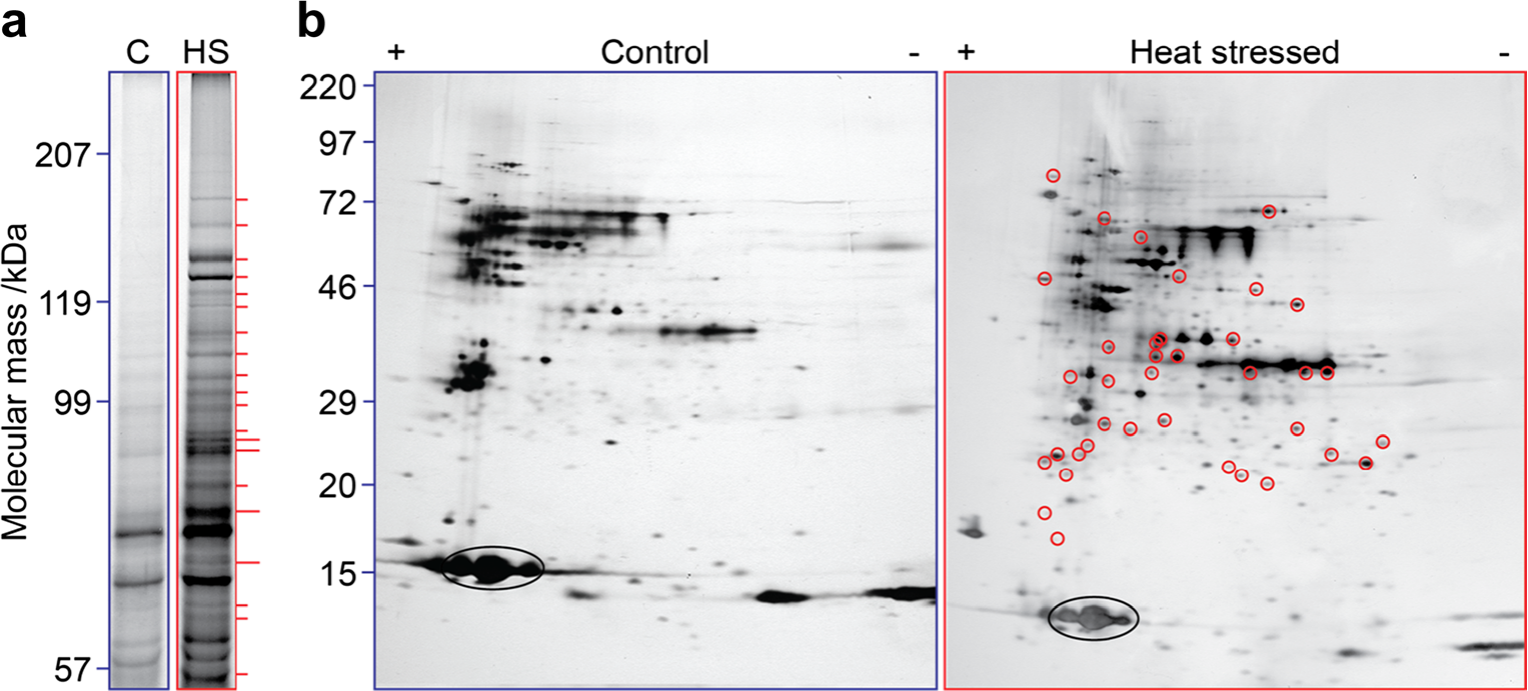
Identification of HSP16.6 interactors. **a** SDS-PAGE separation of proteins recovered in association with HSP16.6-Strep in cells grown at 30 °C and treated at 42 °C for 2 h plus 1 h recovery at 30 °C to allow sHSP accumulation (control sample, C) or further treated with an additional 30 min at 46 °C (heat-stressed sample, HS). To recover proteins in the high molecular mass range, separation was performed using an 8% acrylamide gel, and the position of molecular mass markers is indicated. Bands that were excised for analysis are annotated with red dashes. Double-width dashes indicate bands that gave hits for proteins associated with protein-folding processes. **b** 2D gel separation of samples prepared as described in **a**. The position of molecular mass markers and the acidic (+) and basic (−) sides of the silver-stained 2D gels are indicated. Spots that were excised and yielded the reported data are annotated with red circles (right panel). The ellipse in each panel indicates the spots due to HSP16.6

The HSP16.6-Strep strain and an isogenic strain carrying wild-type HSP16.6 were subjected to mild heat stress to allow accumulation of the sHSP and then to a short, more severe heat stress to maximize association of thermally unstable proteins with the sHSP. The soluble cell fraction from control and heat-stressed cells of the HSP16.6-Strep and HSP16.6 strains was subjected to Strep-Tactin affinity chromatography and the recovered proteins compared by means of 2D electrophoresis (or, to examine high molecular mass proteins, by using 1D electrophoresis) (Fig. 1). Individual spots or bands unique to proteins affinity-purified with HSP16.6-Strep from the heat stress samples were excised and subjected to MS analysis.

We identified a total of 72 proteins in these experiments which, when combined with others we had identified previously (Basha et al. 2004), expanded to a total of 83. Notably, the proteins were recovered from the soluble fraction, so they do not represent those that underwent excessive aggregation, or associations with membranes and cytoskeletal elements that may have led to partitioning into the pellet. As such, these proteins represent potential sHSP interactors that have been prevented from insolubilization by interaction with HSP16.6. We denote this set of interactors *I*, representing a subset of the genome *G* detectable in our experiments. This allows us to test hypotheses about the features of these interactors to shed light on what distinguishes them from the other proteins in *Synechocystis*. Though many of the interactors have known PPIs, based on cross-referencing to genome-wide yeast-2-hybrid data (Sato et al. 2007) (Supplementary Table 1), notably there are only three described pairwise PPIs within *I*, and all three of these are self-associations. To see if this low count was an artifact from our conservative approach of excluding PPIs that were identified with only one prey clone, we also tested including the latter, which presumably yields more false positives. This increased the number of pairwise PPIs within *I* to 12, including six self-interactions, which is still a small subset of *I*. Consequently, the proteins in *I* appear largely independent of each other in their interaction with HSP16.6, consistent with our affinity-isolate methodology being sensitive to stable interactors.

### Primary- and secondary-structure features of HSP16.6 interactors

**Fig. 2:**
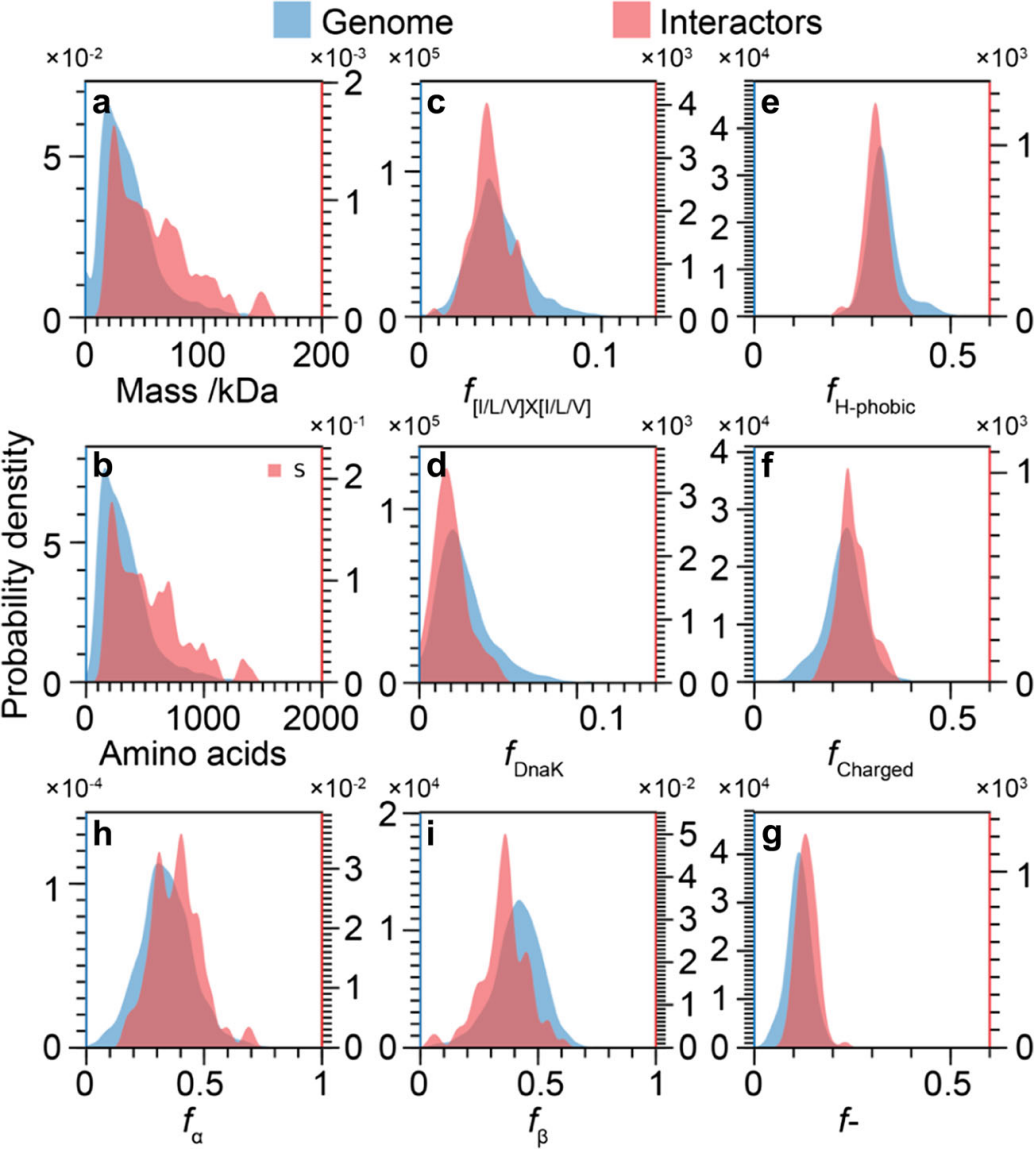
Probability distributions of the statistically significant differences identified in Table 1. **a**, **b** The distributions of protein mass (**a**) and sequence length (**b**) for *I* and *G*. The proteins in *I* are on average approximately 60% larger than those in *G*, both in terms of mass and sequence length. **c**, **d** Distributions of frequencies of [I/L/V]X[I/L/V] motifs (**c**) and DnaK-binding motifs (**d**). Both sequence features are less frequent and more narrowly distributed in *I*. **e**–**g** The fraction of hydrophobic (**e**), charged (**f**), and negative (**g**) residues. Charged residues are more frequent in *I*, which can be attributed to a higher fraction of negatively charged residues and a lower fraction of hydrophobic residues. **h**, **i** Fraction of residues with predominately helical (α and 3_10_, **h**) propensity and β-structure (sheet and turn, **i**). The helix content is higher in *I* than in *G*, and conversely, the β-structure content is lower in *I*. The distributions were normalized such that their integral equals the number of proteins in each set. Consequently, the amplitudes are inversely proportional to the width of the distributions, and the amplitudes of the two distributions in each panel reflect the different sizes of the two sets

We first compared the average mass and sequence lengths of the interactors to the genome. We found that these were very different, with the interactors being about 60% larger on average (Table 1, Fig. 2a, b). While this is informative about the interactor profile of HSP16.6, it also means that the absolute number, *n*_*F*_ of any feature *F*, is likely to be larger for the interactors. To account for this, all subsequent analyses are consequently focused on fractional quantities, *f*_*F*_, which are normalized by sequence length in order to reveal distinctive features for the proteins associated with HSP16.6.

**Table 1:** Comparison of various primary- and secondary-structure features between interactors of HSP16.6 in *Synechocystis* with the wider genome. Mean values obtained for the proteins in *I* and *G*, along with *p* values for the differences between them

Quantity	Interactors, *I*	Genome, *G*	*p* value
*m*/Da	**57,860**	**36,561**	**< 10** ^**−5**^
*n*_aa_	**525**	**336**	**< 10** ^**−5**^
*f*_DNAK_	**0.0198**	**0.285**	**< 10** ^**−5**^
*f*_VQL_	0.000335	0.000274	0.27
*f*_IXI_	0.00349	0.00305	0.15
*f*_[ILV]X[ILV]_	**0.0378**	**0.0426**	**0.002**
pI	5.22	5.63	0.036
*f*_Charged_	**0.252**	**0.230**	**6.0∙10** ^**−5**^
*f*_+_	0.118	0.115	0.24
*f*_−_	**0.134**	**0.114**	**< 10** ^**−5**^
*f*_H-phobic_	**0.309**	**0.331**	**1.0∙10** ^**−5**^
*f*_d_	0.086	0.058	0.015
*f*_β_	**0.355**	**0.415**	**< 10** ^**−5**^
*f*_α_	**0.383**	**0.338**	**3.1∙10** ^*−5*^

Bold text indicates statistically significant differences, defined as *p* < 0.01

We judged that certain sequence motifs might be implicated in the association of interactors with sHSPs. To develop hypotheses for testing, we considered a model in which interfaces that allow the sHSP to self-assemble might be the same as interactor binding sites (Jacobs et al. 2016). In this context, the inter-monomer contact made between the highly conserved “IXI” motif in the C-terminal region and the β4–β8 groove of the ACD has been proposed as an auto-inhibitory interface (Jehle et al. 2010; van Montfort et al. 2001). Theorizing that IXI motifs might mediate contacts with the sHSPs, we therefore asked whether they were differentially represented in the interactors. We also posed this question in a more general form, by searching for motifs matching the requirement [I/L/V]X[I/L/V], which is more encompassing across the breadth of sHSPs (Poulain et al. 2010). Furthermore, we searched for VQL motifs, as this corresponds to the specific manifestation of the “IXI” in HSP16.6. Comparing the fractional abundance of these motifs (*f*_IXI_, *f*_[ILV]X[ILV]_, *f*_VQL_, respectively) between the interactors and the genome, we found there to be no meaningful difference for IXI and VQL, but the general form [I/L/V]X[I/L/V] was significantly under-represented in the interactors (Table 1, Fig. 2c).

sHSPs are thought to transfer interactors to the DnaK (HSP70 in eukaryotes) system for ATP-dependent refolding (Haslbeck and Vierling 2015). We therefore hypothesized that the presence of DnaK-binding motifs (Rudiger et al. 1997), which mediate association with this downstream chaperone, might be different between the interactors and the genome. We found the fractional abundance of DnaK motifs (*f*_DnaK_) to be > 30% lower in the interactors (Table 1, Fig. 2d).

We next considered electrochemical properties of the proteins. The difference in pI between the interactors and genome was just outside our significance criterion (*p* = 0.036 > 0.01). However, when examining the fraction of charged residues (*f*_Charged_), we discovered it to be higher in the interactors. By investigating negatively and positively charged residues separately (*f*_−_ and *f*_+_, respectively), we found this difference to be due to the former, with negatively charged residues > 16% more abundant in the interactors. Conversely, the genome contains a higher fractional abundance of hydrophobic residues (*f*_H-phobic_) (Table 1, Fig. 2e–g).

Lastly, we asked whether predicted secondary structure differed between the two sets. The fraction of residues in disordered regions (*f*_*d*_) is insignificantly higher in the interactors, albeit very near our threshold (*p* = 0.015 ≈ 0.01). For the structured regions, on average, the interactors had a higher fraction of residues in helices (*f*_α_) and lower fraction in β-structures (*f*_β_), compared to the proteins in the wider genome (Table 1, Fig. 2h, i).

### Functional classification of HSP16.6-associated proteins

**Fig. 3:**
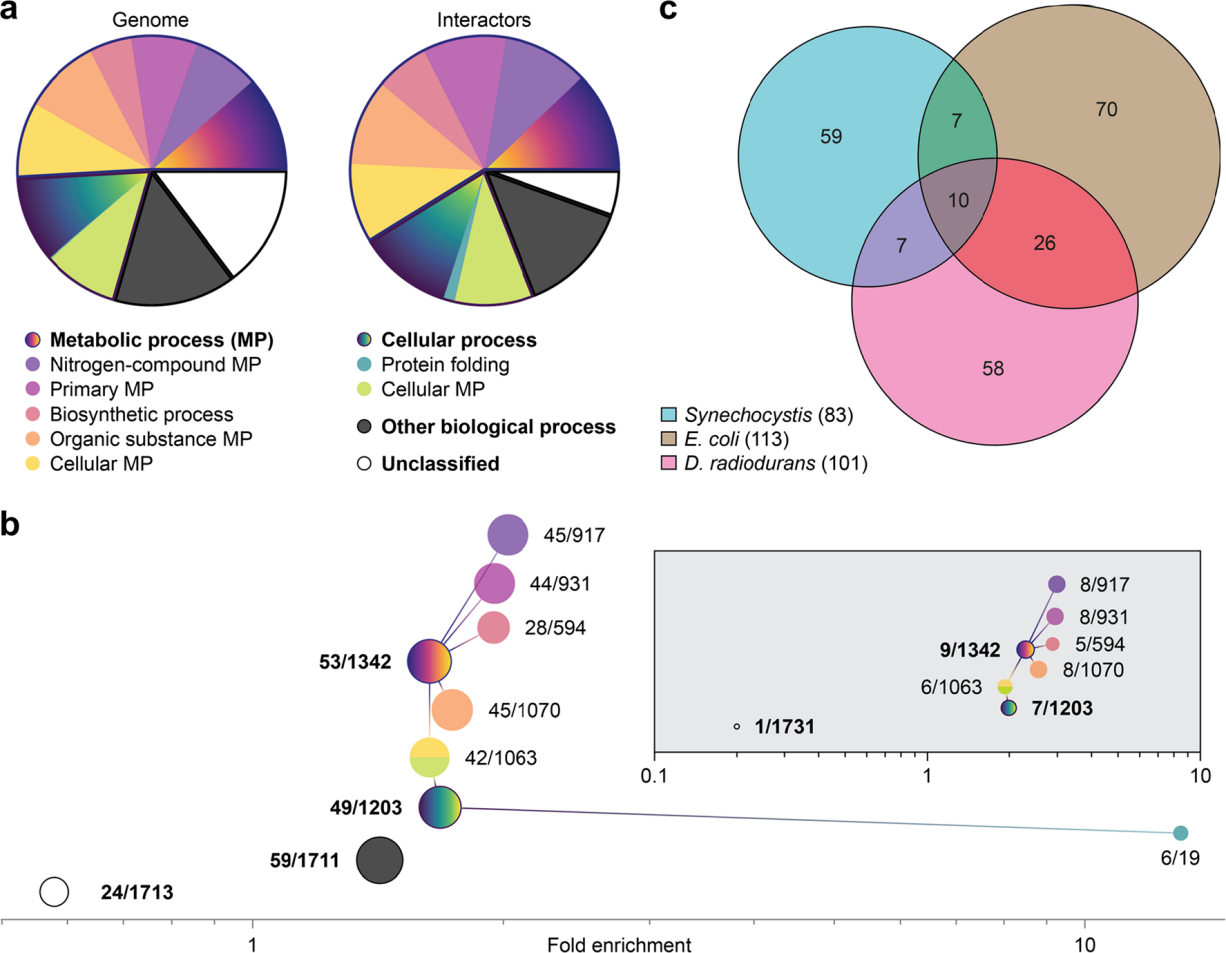
Classification of proteins involved in different gene-ontology annotations of biological processes. **a** Pie charts show the extent of different classes in *I* and *G*. The most fundamental classes have labels in bold face. Note that “cellular metabolic process” belongs to both “metabolic process” and “cellular process” and is therefore represented by two colors. **b** Enrichment within *I* of proteins taking part in the various biological processes. Circle areas reflect the number of proteins in *I*, and numbers indicate proteins in *I* and *G*. *I* contains a smaller fraction of unclassified proteins than *G*, and all classes are somewhat enriched in *I*. Proteins involved in protein folding are enriched thirteen-fold, with 6 of the 19 such proteins known being found among the interactors. Inset: Same analysis performed for the 10 overlapping proteins from the analysis in (**c**). In all featured classes, the fold-enrichment is higher. **c** Venn diagram showing the overlap of sHSP interactor ranges from *Synechocystis*, *E. coli*, and *D. radiodurans*. Note that, with the exception of the intersection of the three sets, all areas of the diagram reflect the number of elements within

Where possible, interactors were classified according to their gene-ontology annotation into either “metabolic process,” “cellular process,” or “other biological process.” Many proteins were assigned to multiple classes, and 15 proteins could not be matched to the reference list and were added to the set of unclassified proteins, which then comprised 24 proteins. This classification yielded different distributions of processes in *I* and *G* (Fig. 3a), indicating that HSP16.6 has an interaction profile that reflects the biological function of its interactors. To quantify the differences, we calculated the overrepresentation of proteins involved in the various biological processes (Fig. 3b). The data reveal statistically significant enrichment of proteins ascribed to certain biological processes in the interactors, suggesting that HSP16.6 makes function-specific interactions. The most striking association was for proteins involved in protein folding, with 6 out of the 19 known such proteins being found in *I* (Table 2), corresponding to a thirteen-fold enrichment.

**Table 2:** The six interactors of *Synechocystis* HSP16.6 annotated as belonging to the “protein folding” category

Gene	UniProt ID	Name
sll0058	Q55154	DnaK 1
sll0170	P22358	DnaK 2
sll1932	P73098	DnaK 3
slr2076	Q05972	60 kDa chaperonin 1
sll0533	Q55511	Trigger factor (TF)
slr1251	P73789	Peptidyl-prolyl cis-trans isomerase

To compare HSP16.6-interactors with those identified in other prokaryotes, we cross-referenced our list with those reported as IbpB interactors in *E*. *coli* (Fu et al. 2013), and HSP20.2 in *D. radiodurans* (Bepperling et al. 2012) (Fig. 3c). There were unique orthologs for 17 HSP16.6 interactors among the 113 IbpB interactors and 17 for the 101 HSP20.2 interactors. The overlap between IbpB and HSP20.2 interactors was larger still, comprising 36 unique orthologs. A total of 10 proteins were found in all three sets of interactors. Notably, these overlaps are much larger than one would expect by chance (approximately 3 for each pairwise overlap, and fewer than 1 for the triple overlap). Interestingly, these proteins were also diverse, spanning multiple biological processes, with only one eluding classification (Table 3, Fig. 3b inset). With the exception of the “protein folding” and “other biological process,” which were not represented at all in this subset, all categories were even more overrepresented than in the complete list of HSP16.6 interactors. We note that the small number of proteins precluded low *p* values for the levels of enrichment for the individual categories. Taken together, they nonetheless indicate that the enrichment pattern seen for the *Synechocystis* interactors is particularly prominent for the interactors that are common for all three sHSPs, with the striking exception of the protein-folding interactors, which might be a species- or sHSP-specific phenomenon, or the result of differences in the methods used for recovering interacting proteins.

**Table 3:** Proteins that we associated to all three of HSP16.6 (*Synechocystis*), IbpB (*E. coli*), and HSP20.2 (*D. radiodurans*). The GO annotations for biological processes are coded as follows: metabolic process (MP), cellular process (CP), nitrogen-compound metabolic process (NCMP), primary metabolic process (PMP), biosynthetic process (BP), organic substance metabolic process (OSMP), cellular metabolic process (CMP), and unclassified (U). In some cases, two distinct IbpB or HSP20.2 interactors would correspond to an HSP16.6 interactor, in which case, both UniProt IDs were included in the table

*Synechocystis* gene	UniProd ID *Synechocystis* *E. coli* *D. radiodurans*	Name	GO biological process
sll0018	Q55664 G64976n NP_295312.1	Fructose-bisphosphate aldolase, class II	MP, CP, NCMP, PMP, OSMP, CMP
sll1099	P74227 NP_289744.1, pdb|1EFC|A NP_295522.1	Elongation factor Tu	MP, CP, NCMP, PMP, BP, OSMP, CMP
sll1180	P74176 NP_287490.1 NP_295291.1	Toxin secretion ABC transporter ATP-binding protein	CP, NCMP, PMP, OSMP
sll1326	P27179 CAA23519.1 NP_294424.1	ATP synthase alpha chain	MP, CP, NCMP, PMP, BP, OSMP, CMP
sll1787	P77965 AAC43085.1 NP_294636.1	RNA polymerase beta subunit	MP, CP, NCMP, PMP, BP, OSMP, CMP
sll1789	P73334 NP_290619.1 NP_294635.1	RNA polymerase beta’ subunit	MP, CP, NCMP, PMP, BP, OSMP, CMP
sll1818	P73297 CAA37838.1 NP_295851.1	RNA polymerase alpha subunit	MP, CP, NCMP, PMP, BP, OSMP, CMP
sll1841	P74510 NP_285811.1, NP_286443.1 NP_293809.1, NP_293979.1	Pyruvate dehydrogenase dihydrolipoamide acetyltransferase component (E2)	MP
slr0542	P54416 NP_286179.1 NP_295695.1	ATP-dependent protease ClpP	MP, NCMP, PMP, OSMP
slr1105	P72749 NP_289127.1 NP_294922.1	GTP-binding protein TypA/BipA homolog	U

## Discussion

Here, we have examined the properties of 83 proteins that associate in vivo with HSP16.6 under conditions of heat stress. Given that the proteins were obtained from the soluble supernatant after centrifugation, they are likely to under-represent membrane- and cytoskeleton-associated proteins. Furthermore, as our experiment involves affinity pull-downs, these interactors are inevitably restricted to those that form interactions that are stable on the timescale of the experiment. In the context of the model proposed for sHSPs wherein they display both a low-affinity mode with high capacity, and a high-affinity mode with low capacity (McHaourab et al. 2009), our interactors are likely representative of the latter. Notwithstanding these potential biases of the experiment, we have shown that the interactors were on average larger than the proteins in the genome, have a distinct electrochemical profile, an increased fraction of helical secondary structure, and a lower fraction of [I/L/V]X[I/L/V] and DnaK-binding motifs.

We observed that HSP16.6 preferentially binds longer, more massive, proteins. This is in agreement with analysis of sHSP interactors *E. coli* and *D. radiodurans* (Fu et al. 2014) and is interesting in light of recent data noting that thermally unstable proteins in cells are typically longer than those that are stable (Leuenberger et al. 2017). Longer proteins might therefore be overrepresented in the interactors by virtue of being more likely to be destabilized by the heat-shock condition assayed here. Alternatively, or in addition, it is possible that longer proteins, by virtue of having more binding sites, might be held tighter by the sHSPs. This would stem from avidity effects resulting from the multivalency of sHSP oligomers (Hilton et al. 2013), similar to observations made for other molecular chaperones (Huang et al. 2016; Saio et al. 2014).

Upon considering amino acid motifs and composition, we found a lower fraction of [I/L/V]X[I/L/V] motifs in the interactors. This suggests that the β4–β8 groove, which binds this motif intra-molecularly in sHSP oligomers (Basha et al. 2012; Hilton et al. 2013), is not the binding site for these stable interactors. However, this does not preclude the β4–β8 groove being a site for low-affinity, or transient, interactions. This is consistent with the observation that the excised ACD can display potent chaperone activity (Cox et al. 2016; Hochberg et al. 2014). We also identified an overabundance of charged and, in particular negatively charged, residues in the interactors. A preponderance of charged residues was also observed for sHSP interactors in *E. coli* and *D. radiodurans* (Fu et al. 2014). Notably, aspartates have been shown to be enriched in thermally unstable proteins (Leuenberger et al. 2017), again hinting that thermal stability could be a key attribute for recognition by sHSPs. It is also interesting to consider the electrochemical profile of the sHSPs themselves, which have an overabundance of charged residues in the ACD and C-terminal region (Kriehuber et al. 2010). As such, it is possible that there may be charge-complementarity aspects to binding.

The depletion of DnaK-binding motifs in the HSP16.6 interactors is striking, particularly when considering that DnaK is able to release interactors from the complexes made with HSP16.6. This suggests that the DnaK-binding motif is not responsible for the recognition events that mediate interactor transfer between the chaperones. Instead, the DnaK-binding motif may be more reflective of DnaK’s holdase, rather than refoldase activity. In this way, proteins that are not protected by the sHSPs are captured by HSP70 instead (Mayer and Bukau 2005). The interactors are also enriched in α-helical propensity and depleted in β-structure. It is possible that, based on the observation that there is little cooperativity in the folding of β-sheets (Wu and Zhao 2001), this may be reflective of physico-chemical differences in re- or unfolding.

Gene-ontology analysis demonstrates that, while capable of associating with many interactors, HSP16.6 nonetheless does so with statistically significant specificity, evidenced by varying enrichments for different biological processes. This observation is validated by the overlap between *Synechocystis*, *E. coli*, and *D. radiodurans* sHSP interactors. The notion that sHSPs have specific interactors in the cell also extends to eukaryotes, where different sHSPs found in the same cellular compartment have differing interactor profiles (Fleckenstein et al. 2015; McLoughlin et al. 2016; Mymrikov et al. 2017).

The most enriched groups of proteins associated with HSP16.6 were other components of the protein folding machinery. We interpret this as due to HSP16.6 being part of a tightly linked molecular chaperone network (Gong et al. 2009), collaborating to prevent and reverse improper protein interactions in the wider heat-shock response of the cell (Richter et al. 2010). Possibly, these interactions are indirect, captured due to HSP16.6 and other protein-folding components acting on the same substrates. An indirect interaction with protein-folding components could also explain the lack of equivalent proteins in the *E. coli* sHSP interactors (Fu et al. 2013), as the previous report employed covalent-crosslinking and urea solubilization prior to immunoprecipitation. The *D. radiodurans* interactors were identified by a different method, employing ex vivo addition of purified HSP20.2 to cell lysates, prior to heat stress and immunoprecipitation. Given the differences in methodology between these studies, we suggest that those proteins comprising common interactors are highly significant (Table 3).

In sum, our study provides an initial view of the functional interactome of prokaryotic sHSPs and of *Synechocystis* in particular. In addition, the statistical framework we have implemented for examining sequence determinants can be applied to the analysis of the likely future profusion of proteomic data identifying molecular chaperone interactors in cells.

## Electronic supplementary material

ESM 1 (DOCX 28 kb)
