# Supplementary material for: Structural and functional aspects of the interaction partners of the small heat-shock protein in Synechocystis
Source: Cell Stress Chaperones. 2018 Feb 23;23(4):723–32. doi: 10.1007/s12192-018-0884-3 (PMC6045555; doi:10.1007/s12192-018-0884-3)
Supplement: Supplementary file 1 — (DOCX 28 kb) [file 12192_2018_884_MOESM1_ESM.docx]

Supplementary material

**Structural and functional aspects of the interaction partners of the small heat-shock protein in *Synechocystis***

Erik G Marklund^1,†^, Yichen Zhang^2,‡^, Eman Basha^2,§^, Justin L.P. Benesch^1,*^ & Elizabeth Vierling^2,*^

*^1^ Department of Chemistry, Physical & Theoretical Chemistry Laboratory, University of Oxford, Oxford, OX1 3QZ, U.K.*

*^2^ Department of Biochemistry & Molecular Biology, University of Massachusetts,*

*Amherst, MA 01003, U.S.A.*

**Present addresses:**

^†^ Department of Chemistry - BMC, Uppsala University, Uppsala, Sweden;

^‡^ Alorica, Inc., Irvine CA, U.S.A;

^§^ Department of Molecular and Cellular Biology, University of Arizona, Tucson, AZ 85721, U.S.A.

* Correspondence to: justin.benesch@chem.ox.ac.uk, vierling@biochem.umass.edu

**Supplementary Methods**

**Estimating *m* and *pI***

Protein masses were determined directly from the sequence by adding the corresponding amino-acid masses from the Chemical Component Directory, and subtracting the mass corresponding to one water molecule per peptide bond.

pI estimates were also made based on sequence, where every residue and terminus in a protein were first assigned a p*K*_a_ as listed in Supplementary Table 2. The net charge of the protein was then expressed as a function of pH and the p*K*_a_ of its residues. Residues K, R, and H become positive upon protonation, whereas residues D, E, C and Y become negative when deprotonated. The contribution *q_i_* from any such residue to the net charge *q* is thus given by:

$$q_{i}=\left\{ \begin{matrix} \frac{{10}^{{\text{p}K}_{\text{a}}}}{{10}^{\text{pH}}-{10}^{{\text{p}K}_{\text{a}}}}, & \text{(}\text{K}\text{, }\text{R}\text{, }\text{H}\text{)} \\ & \\ -\frac{{10}^{\text{pH}}}{{10}^{\text{pH}}-{10}^{{\text{p}K}_{\text{a}}}}, & \text{(}\text{D}\text{, }\text{E}\text{, }\text{C}\text{, }\text{Y}\text{)} \end{matrix} \right.$$

The net charge is simply the sum of all *q_i_*:

$$q=\sum_{i=1}^{n_{\text{aa}}} q_{i}$$

The pI was determined iteratively, where the pH was initially set to 7.0. If *q* was positive at $\text{pH}=7.0$, the pH was incremented in steps of 0.01 and *q* recalculated until it became ≤0. Conversely, if *q* was initially negative, the pH was decreased in steps of 0.01 until *q* became ≥0. The pI was then taken to be the pH value at which the sign-inversion occurred.

**Supplementary Table 1**

Proteins we identified in this study as interactors of HSP16.6 in *Synechocystis*, with their known PPIs in the genome. Added to this are proteins (*) we had identified as interactors previously (Basha, E. et al, J Biol Chem 279, 7566-75, 2004). None of the PPI partners of a given interactor was also present in the rest of our set of interactors. Three proteins make self-interactions however, and are shown in boldface. Protein names were taken from the Entrez database system.

| Gene | Uniprot ID | Name | PPIs |
| --- | --- | --- | --- |
| sll1789 | P73334 | DNA-directed RNA polymerase subunit beta' |  |
| sll1787 | P77965 | DNA-directed RNA polymerase subunit beta | sll2012, slr1441, sll0687 |
| slr1044 | P73008 | Methyl-accepting chemotaxis protein | slr1618, sll0269, slr0640, slr0171 |
| sll0041 | Q55445 | Putative methyl-accepting chemotaxis protein | sll1225, sll1512, sll0798, sll0269, slr2037, slr1636, slr0474 |
| sll1294 | P73173 | PilJ protein | sll1956 |
| slr1055 | P73020 | Mg-chelatase subunit | slr0653, sll1803 |
| slr0750 | P28372 | Light-independent protochlorophyllide reductase subunit N | sll1292 |
| slr0288 | P77970 | Glutamate–ammonia ligase | slr0744 |
| slr0898 | Q55366 | Ferredoxin–nitrite reductase |  |
| slr0585 | P77973 | Argininosuccinate synthase | sll1292, sll1491, slr0199 |
| sll0169 | H0PFL3 | - | slr1127, slr1629, slr0171, slr1218, slr0533, sll1880 |
| slr0228 | Q55700 | ATP-dependent zinc metalloprotease FtsH 2 |  |
| slr1604 | P72991 | ATP-dependent zinc metalloprotease FtsH 3 |  |
| slr0156 | P74459 | Chaperone protein ClpB 1 | slr0111 |
| slr0659 | P74571 | Oligopeptidase A | sll0696 |
| sll0170 | P22358 | Chaperone protein dnaK2 |  |
| sll0058 | Q55154 | Chaperone protein dnaK1 |  |
| sll1932 | P73098 | Chaperone protein dnaK3 |  |
| slr2076 | Q05972 | 60 kDa chaperonin 1 |  |
| slr0322 | H0PFH5 | - | slr1777 |
| sll0043 | H0PID4 | - | ssl2296, slr1645, sll1691 |
| sll1672 | H0PI99 | - | ssl0241, slr0423 |
| sll1561 | P74275 | Delta-1-pyrroline-5-carboxylate dehydrogenase |  |
| sll1178 | P74178 | Uncharacterized protein |  |
| slr1463 | P28371 | Elongation factor G 1 |  |
| **sll1841** | P74510 | Dihydrolipoamide acetyltransferase component (E2) of pyruvate dehydrogenase complex | ssr1256, sll0931, sll1841, sll1721, slr1096, ssr0536 |
| sll1031 | P72758 | Carbon dioxide concentrating mechanism protein | sll0905, sll1489, sll1032, slr1347 |
| sll1180 | P74176 | HlyB family | sll0226 |
| sll0533 | Q55511 | Trigger factor | sll0507 |
| slr0335 | Q55544 | Phycobiliprotein ApcE | ssl3692, slr1596 |
| slr2088 | P73913 | Acetohydroxy acid synthase | slr0630 |
| sll1326 | P27179 | ATP synthase subunit alpha |  |
| slr0940 | P74306 | Zeta-carotene desaturase |  |
| **slr0869** | H0PM77 | - | sll1574, slr0869 |
| sll1033 | H0PI64 | - | ssl2667 |
| sll1583 | P73196 | DNA ligase |  |
| slr6071 | Q6YRT3 | Slr6071 protein (Slr6012 protein) |  |
| sll0923 | P72877 | Exopolysaccharide export protein | ssr0692, sll1653, sll1472 |
| sll1770 | P73627 | Uncharacterized protein | slr0211 |
| slr0758 | P74646 | Circadian clock protein kinase KaiC |  |
| sll0245 | P73886 | Sll0245 protein |  |
| slr1643* | Q55318 | Ferredoxin–NADP reductase | sll0415 |
| slr0049 | H0PG67 | - |  |
| sll0851 | F7UPA9 | - | slr1655, slr0341 |
| sll1184 | P72849 | Heme oxygenase 1 |  |
| slr1742 | H0PM42 | - |  |
| slr1898 | P73326 | Acetylglutamate kinase |  |
| slr0164 | P74466 | Putative ATP-dependent Clp protease proteolytic subunit-like |  |
| slr0542 | P54416 | ATP-dependent Clp protease proteolytic subunit 1 | sll0534, slr7037 |
| sll0998 | P73123 | Probable RuBisCO transcriptional regulator |  |
| sll1626 | P73722 | Transcription regulator LexA |  |
| slr0657 | P74569 | Aspartokinase (Aspartate kinase) |  |
| sll1099 | P74227 | Elongation factor Tu |  |
| sll1261 | P74070 | Elongation factor Ts |  |
| sll1098 | P74228 | Elongation factor G 2 | sll1293 |
| sll1342 | P80505 | Glyceraldehyde-3-phosphate dehydrogenase 2 |  |
| slr0965 | P72856 | Beta sliding clamp |  |
| sll0569 | P74737 | Protein RecA |  |
| **sll0947** | P74518 | Ribosome hibernation promotion factor | sll1773, sll0947 |
| sll1545 | P74665 | Glutathione S-transferase |  |
| sll0018 | Q55664 | Fructose-bisphosphate aldolase class 2 |  |
| sll1931 | P77962 | Serine hydroxymethyltransferase |  |
| slr0549 | Q55512 | Aspartate-semialdehyde dehydrogenase |  |
| sll0899 | Q55504 | Bifunctional protein GlmU |  |
| slr1984 | P74142 | 30S ribosomal protein S1 homolog B |  |
| sll0144 | P74457 | Uridylate kinase |  |
| slr1198 | H0PKZ1 | - | sll1574, sll1404 |
| sll1316 | P26290 | Cytochrome b6-f complex iron-sulfur subunit 2 |  |
| slr0520 | Q55843 | Phosphoribosylformylglycinamidine synthase subunit PurQ |  |
| slr0244 | H0PI00 | - |  |
| slr0552 | H0PIM1 | - |  |
| sll0617 | Q55707 | Uncharacterized protein |  |
| sll1218 | P74029 | Ycf39 protein | sll0177, sll0208, slr0452 |
| slr1105* | P72749 | GTP-binding protein TypA/BipA homolog |  |
| slr1329* | P26527 | ATP synthase subunit beta | slr0546, slr1648 |
| slr1356* | P73530 | 30S ribosomal protein S1 homolog A | slr0923 |
| sll1818* | P73297 | DNA-directed RNA polymerase subunit alpha |  |
| sll1284* | P73192 | Serine esterase |  |
| sll0643* | P72955 | Urease accessory protein UreG |  |
| sll1669* | P72796 | Shikimate kinase | sll2005, slr1837 |
| slr2024* | H0PKN1 | - |  |
| slr1251* | P73789 | Peptidyl-prolyl cis-trans isomerase |  |
| slr0992* | P74516 | Putative tRNA (cytidine(34)-2'-O)-methyltransferase |  |

**Supplementary Table 2**

pKa values for amino-acid residues and termini as obtained from http://pepcalc.com.

| Residue | p*K*_a_ |
| --- | --- |
| R | 12.4 |
| K | 10.5 |
| H | 6.00 |
| D | 3.86 |
| E | 4.25 |
| C | 8.33 |
| Y | 10.0 |
| N-terminus | 9.69 |
| C-terminus | 2.34 |
